# Successful treatment of onychomatricoma with minimally invasive surgical procedures based on its pathogenesis: A case report

**DOI:** 10.1111/1346-8138.17088

**Published:** 2024-01-11

**Authors:** Julia Miyamoto, Yuhei Kawashima, Michitaro Hayakawa, Takayuki Fusumae, Tetsuya Yoshida, Masataka Saito

**Affiliations:** ^1^ Department of Dermatology Keio University School of Medicine Tokyo Japan; ^2^ Department of Dermatology Tokyo Women's Medical University, Adachi Medical Center Tokyo Japan; ^3^ Department of Dermatology Tokyo Saiseikai Central Hospital Tokyo Japan; ^4^ Department of Dermatology Tokyo Dental College Ichikawa General Hospital Ichikawa Japan; ^5^ Division of Dermatology National Hospital Organization Tokyo Medical Center Meguro Japan

**Keywords:** CD13, fibroepithelial tumor, onychomatricodermis, onychomatricoma, surgical treatment

## Abstract

Onychomatricoma is a rare, fibroepithelial tumor of the nail. Although it is benign, unnecessary and excessive treatment, such as extensive or total removal of the nail matrix, has been reported in the past. Recently, it was speculated that onychomatricoma is derived from onychomatricodermis, the dermal stroma of the nail matrix. Excision of the stromal rather than the epithelial component of the tumor is important. However, since the boundary between the normal and diseased stroma is usually unclear, minimal excision at the base of the tumor projection should be sufficient. We report a case of onychomatricoma and suggest a method of surgical treatment that would minimize postoperative deformity of the nail plate.

## 1 INTRODUCTION

Onychomatricoma is a rare, benign tumor of the nail, first described by Baran and Kint in 1992.^1^ Onychomatricoma is clinically characterized by xanthonychia, increased nail thickness, increased transverse curvature of the nail, and splinter hemorrhages.^2^ Onychomatricoma is a fibroepithelial tumor that histologically arises in the nail matrix and forms a hole in the nail plate which accommodates its epithelial digitations.^3^

Here, we report a case of onychomatricoma in a Japanese male and suggest a desirable method of surgical treatment to minimize postoperative deformity of the nail plate, in light of a recent report on the pathogenesis of onychomatricoma.^4^

## 2 CASE REPORT

**FIGURE 1:**
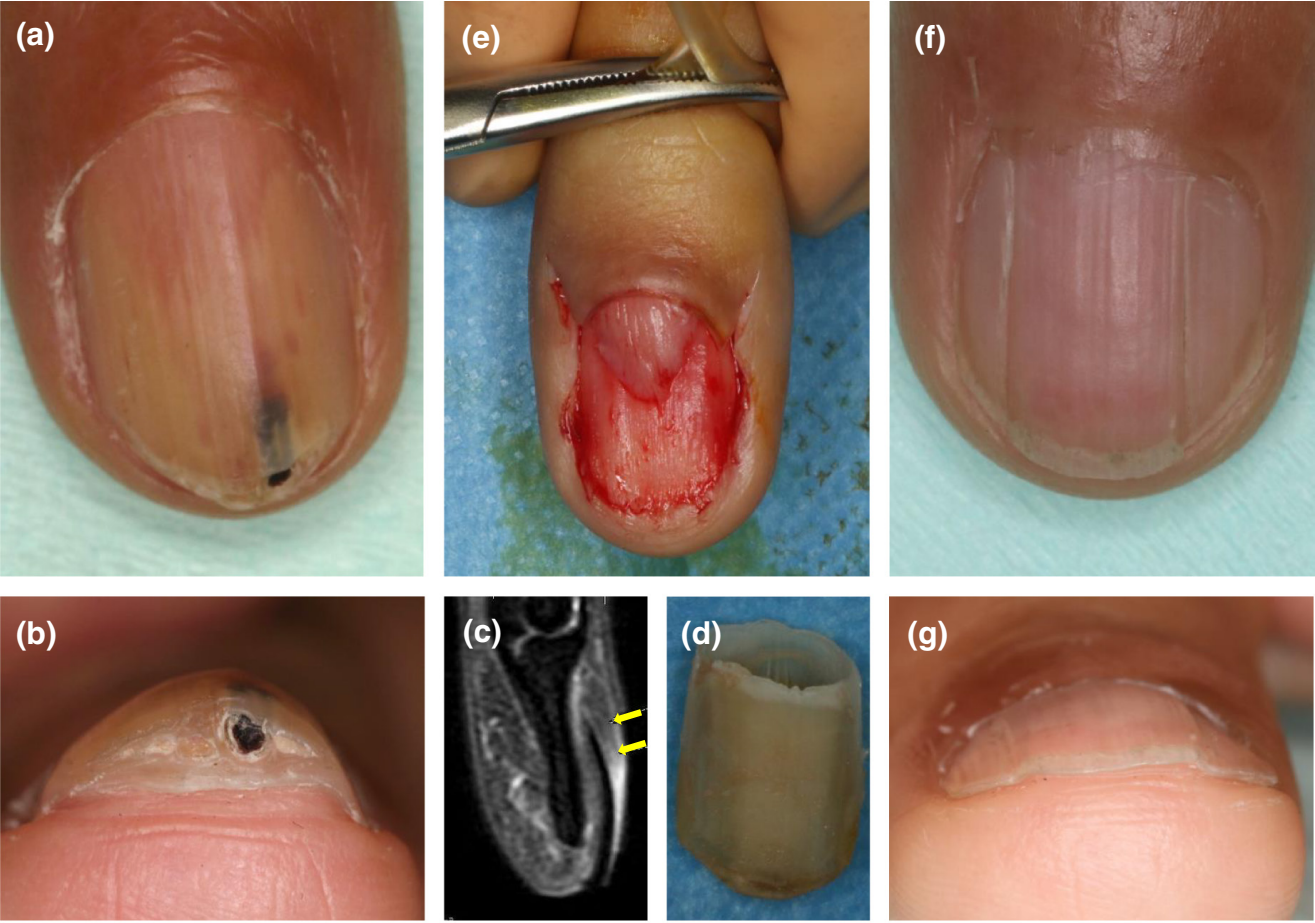
(a, b) The nail plate showed multiple small cavities partially containing hematomas. (c) T2‐weighed magnetic resonance imaging demonstrated a lesion at the nail root. (d, e) Inside the nail plate was a whitish tumor extending from the nail matrix. (f, g) 1 year later, a nearly normal‐looking nail plate had been formed.

A Japanese male in his 40s visited our hospital with a complaint of nail deformity of the right ring finger with hemorrhages, which had been occurring for several years. Physical examination revealed that the nail plate was yellowish and thickened toward its center, and observation from the distal edge showed multiple small cavities inside the nail plate, some of which contained hematomas (Figure 1a,b).

Clinically, we suspected a neoplastic lesion of the nail unit, and T2‐weighed magnetic resonance imaging demonstrated a lesion of high signal intensity at the nail root in the sagittal section which extended approximately 10 mm into the nail plate (Figure 1c). Nail clipping examination was attempted and histological examination showed multiple cavities containing serous material in the nail plate, strongly supporting the diagnosis of onychomatricoma.^5^

**FIGURE 2:**
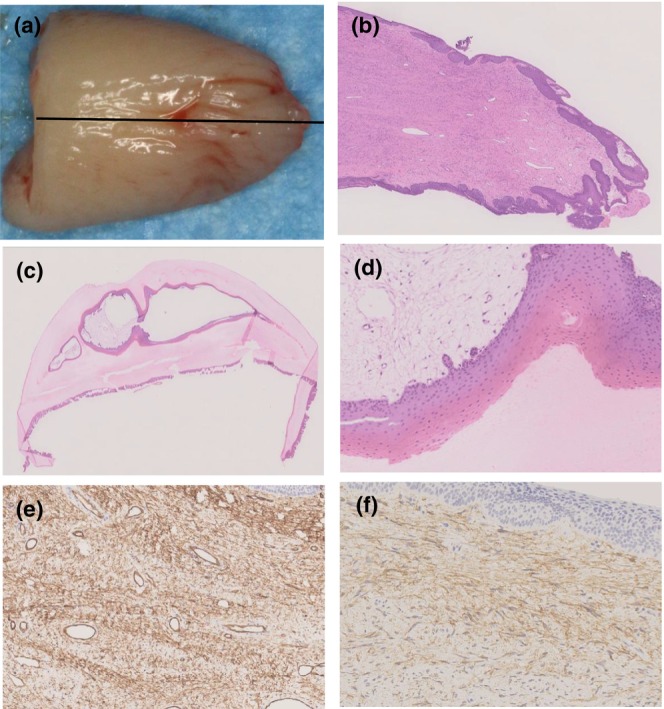
(a) A broadly basal, stalked, whitish tumor was seen from the nail matrix. The tip of the tumor was deeply embedded in the nail matrix. A longitudinal section of the tumor was made (black line). (b) The lesion was a fibroepithelial tumor with proliferation of hyperkeratotic squamous cells and numerous spindle‐shaped cells between collagen fibers (×25). (c, d) Multiple cavities inside the nail plate were covered by epithelium, lacking granular layers and showing keratinizing transition layers stained darkly with eosin (c×12.5, d×100). (e) Spindle‐shaped cells found in the dermis were strongly positive for CD34 (×130). (f) The spindle‐shaped cells were largely positive for CD13 (×250).

Under local anesthesia, the nail plate was carefully and gently avulsed, revealing a whitish projection of the tumor extending from the area corresponding to the nail matrix (Figure 1d,e). Since onychomatricoma is a benign tumor, priority was given to minimizing postoperative nail deformity, preserving nail function as much as possible, and increasing the patient's satisfaction with the cosmetic appearance of the nail. Accordingly, an incision was made to the margin of the base of the lesion to accomplish minimum surgical removal (Figure 2a). Histologically, the lesion was a fibroepithelial tumor composed of epithelial and stromal components, with proliferation of squamous cells with hyperkeratosis and numerous spindle‐shaped cells between collagen fibers as well as scattered mast cells (Figure 2b). Multiple cavities observed inside the avulsed nail plate were covered by the epithelium, lacking granular layers, and showing keratinizing transition layers stained darkly with eosin, resembling that of the nail matrix (Figure 2c,d). Immunostaining showed that spindle‐shaped cells found in the dermis of the lesion were strongly positive for CD34 (Figure 2e). In addition, they were positive for CD13 overall (Figure 2f), but partly positive for CD10. Based on the above histological findings and immunostaining results, we made a final diagnosis of onychomatricoma.

After 1 year, when the patient visited us for postoperative follow‐up, a nearly normal‐looking nail plate had been formed with slight trace changes consistent with the site of the tumor. The patient was extremely satisfied with the result from the cosmetic point of view (Figure 1f,g).

## 3 DISCUSSION

Onychomatricoma is known to present with characteristic clinical and histological features, and its diagnosis and treatment require an adequate understanding of the anatomy of the nail unit and its pathogenesis and pathophysiology.^2^, ^3^ Onychomatricoma is a tumor that originates in the nail matrix, which often forms numerous finger‐like projections and extends into cavities of the thickened nail plate. Although onychomatricoma may appear to be a neoplastic growth of the nail matrix, it is actually a fibroepithelial tumor composed of epithelial and stromal components. Recently, Lee et al. proposed the concept of onychodermis for a specialized nail mesenchyme containing CD10‐positive fibroblasts just below the nail matrix and nail bed. The authors stated that the interaction of this peculiar mesenchyme with the nail matrix epithelium is significant for the nail plate formation.^6^ They also speculated that nail matrix‐like epithelium in onychomatricoma may be induced by a mesenchymal tumor occurring in the nail matrix onychodermis.^4^ Interestingly, the dermal stroma of the nail matrix is rich in CD13‐positive spindle‐shaped cells, which they called onychomatricodermis, and is distinct from that of the nail bed.^4^ It has been demonstrated that CD13 is expressed in a variety of tissues and organs, including sites of epithelial‐mesenchymal interactions such as the papilla of the hair follicle.^7^ Actually in that report, spindle‐shaped cells in the stroma were strongly positive for CD13 in all three cases of onychomatricoma, suggesting that they were derived from onychomatricodermis.^4^ The results of immunostaining in our case also support their hypothesis.

Based on the hypothesis proposed by Lee et al., onychomatricoma seems to be a mesenchymal tumor that accompanies reactive hyperplasia of the matrix epithelium.^4^ Therefore, it may be more important to pay attention to excision of the stromal rather than the epithelial component of the tumor in surgical treatment. However, since the boundary between the normal and diseased stroma is usually unclear, it is difficult to determine how much of the stroma should be excised. On the other hand, onychomatricoma is a benign tumor, and even if a portion is left behind and the possibility of recurrence remains, a wide excision of the nail matrix that would result in nail plate disfigurement should be avoided, and minimal excision at the base of the tumor projection would be sufficient. Minimally invasive surgical treatment for onychomatricoma, as performed in our case, is desirable to preserve nail function and increase patient satisfaction with the postoperative cosmetic appearance of the nail plate. Unnecessary and excessive treatment, such as extensive or total removal of the nail matrix, as reported in the past, should be avoided.^8^, ^9^, ^10^

## CONFLICT OF INTEREST STATEMENT

None declared.
